# Machine Learning for Classification in Lung Cancer Using Routine Clinical and Laboratory Data

**DOI:** 10.1245/s10434-025-18747-y

**Published:** 2025-12-03

**Authors:** Chang Liu, YuLin Liao, Dongsheng Wang, Jie Yang, Liwei Zhao, Xiaoling Liu, Zuo Wang, Lichun Wu

**Affiliations:** Department of Clinical Laboratory, Sichuan Clinical Research Center for Cancer, Sichuan Cancer Hospital & Institute, Sichuan Cancer Center, University of Electronic Science and Technology of China, Chengdu, China

**Keywords:** Lung cancer, Classification, Diagnosis, Machine learning, Calculator

## Abstract

**Background:**

Accurate pathological classification of lung cancer is essential for informing treatment strategies. However, invasive biopsy procedures are not feasible for high-risk patients or those with inaccessible lesions. This study aimed to develop a machine learning model utilizing routine clinical and laboratory data for classification of non-invasive lung cancer.

**Methods:**

Data from patients admitted to Sichuan Provincial Cancer Hospital were retrospectively analyzed. Key features were determined using LASSO and Boruta algorithms. Four machine learning models, including logistic regression, extreme gradient boosting (XGBoost), categorical boosting (CatBoost), and random forest (RandomForest), were trained and optimized through five-fold cross-validation. Model performance was assessed using the area under the receiver operating characteristic curve (AUC), accuracy, and F1 score. An online calculator was developed using R Shiny for clinical deployment.

**Results:**

A total of 1122 patients with lung cancer were included and randomly assigned to the training and test sets. In the training set, 16 features were incorporated into the models. The RandomForest model demonstrated superior performance compared with the other models, achieving an AUC of 0.999, an accuracy of 0.984, and an F1 score of 1.000. Notably, sex and tumor markers were identified as significant predictors. In the test set, the RandomForest model attained a micro-averaged AUC of 0.969 and macro-averaged AUC of 0.940. Sensitivity and specificity varied from 0.667 to 0.995 across subtypes. A web-based tool was implemented to facilitate real-time clinical application (https://nkuwangkai.shinyapps.io/lung-cancer-v1/).

**Conclusion:**

This study presented a robust, non-invasive machine learning model for lung cancer subtype classification, addressing critical gaps in clinical practice for biopsy-ineligible patients. A web-based calculator was developed to facilitate clinical application. Nonetheless, future multicenter validation is warranted to expand the generalizability of this model and promote adoption in diverse healthcare settings.

**Supplementary Information:**

The online version contains supplementary material available at 10.1245/s10434-025-18747-y.

Lung cancer is the second most prevalent type of cancer globally and is the leading cause of cancer-related mortality, with an overall 5-years survival rate of <20%.^1^ Early diagnosis and appropriate treatment of lung cancer can significantly enhance patient survival rates by 20%.^2^ Lung cancer can be divided into small-cell lung cancer (SCLC) and non-small-cell lung cancer (NSCLC), with NSCLC accounting for approximately 80% of cases. NSCLC encompasses subtypes such as squamous cell carcinoma (SCC) and adenocarcinoma (ADC), among others. Histopathological analysis remains the gold standard for histological classification and plays an essential role in determining treatment strategies.^3^ However, some lesions are located in anatomically inaccessible sites, and certain patients have an elevated risk of biopsy-related complications, including pneumothorax, hemorrhage, pleural reactions, and even tumor implantation. Such challenges complicate disease diagnosis and treatment. Furthermore, studies have indicated that 3–10% of epidermal growth factor receptor (EGFR)-mutant NSCLC cases may transform into SCLC, leading to rapid disease progression.^4^ The identification of mutation sites in NSCLC heavily relies on pathological evaluation, which introduces significant diagnostic uncertainty. Consequently, accurate pathological classification remains an urgent challenge.

In recent years, advancements in radiomics, proteomics, and metabolomics have offered promising non-invasive alternatives for tumor characterization. Radiomics, in particular, facilitates comprehensive three-dimensional analysis of entire tumors, overcoming the limitations associated with biopsy and enhancing diagnostic accuracy.^5,6^ However, most radiomics studies are conducted at single centers and lack external validation. Furthermore, no standardized operational protocols have been established for radiomics techniques, leading to variability across sites. Proteomics and metabolomics represent powerful methodologies for the classification of lung cancer.^7,8^ Nevertheless, proteomics requires expensive mass spectrometers and antibody chips and involves financial implications for dynamic monitoring. Such limitations pose significant challenges, particularly in economically disadvantaged regions. In metabolomics analysis, metabolite levels are influenced by factors such as dietary intake, circadian rhythms, and medications. Moreover, the complexity of metabolic pathways necessitates bioinformatics expertise to construct network models, complicating clinical interpretation. Alternative approaches are being explored to overcome these limitations, such as the utilization of serological tumor markers.

Serological markers play a pivotal role in the diagnosis,^9^ staging,^10^ and evaluation of mutational transformations^11^ in lung cancer. These markers are essential for distinguishing between various types of lung cancer and for assessing disease progression and therapeutic response. In the context of SCLC, neuron-specific enolase (NSE) and pro-gastrin-releasing peptide (ProGRP) are particularly pertinent. NSE is a well-established marker for neuroendocrine tumors, with elevated levels frequently observed in patients with SCLC, thereby providing critical information for diagnosis and treatment monitoring.^12^ ProGRP has demonstrated promising results as a marker for SCLC; studies have reported a strong correlation between proGRP and tumor stage and treatment response. Hence, proGRP represents a reliable predictor of disease progression.^11^ Carcinoembryonic antigen (CEA) and cytokeratin 19 fragment (CYFRA21-1) are commonly associated with NSCLC.^13^ CEA is often employed as a prognostic marker in ADC, with elevated levels indicating a higher risk of recurrence and poor overall survival. CYFRA21-1 is a marker frequently elevated in SCC and is utilized to assess disease progression and therapeutic response. Furthermore, SCC antigen (SCCA) is primarily used in the diagnosis and monitoring of SCC.^14^ These markers are low cost and used, but single metrics cannot accurately identify tumor subtypes. These markers could be integrated into a comprehensive tool for effectively screening lung cancer subtypes. Notably, linear regression models are inadequate for aggregating high-dimensional features, whereas machine learning algorithms are specifically designed to address this challenge.

Consequently, some patients with a high suspicion of lung cancer are unable to undergo comprehensive histopathological evaluation, as existing tools for lung cancer classification do not adequately meet clinical needs. This study aimed to address these issues by developing a lung cancer subtype prediction model based on machine learning algorithms, integrating clinical features and serological lung cancer markers.

## Materials and Methods

### Study Design and Participants

The single-center retrospective cohort included patients with lung cancer admitted consecutively to Sichuan Provincial Cancer Hospital between November 2023 and June 2024. In addition, patients with lung cancer admitted to The First Affiliate Hospital of Chengdu Medical College from February 2020 to August 2023 served as a supplementary cohort. This study was approved by the ethics committee of Sichuan Provincial Cancer Hospital (No. SCCHEC-02-2024-130). This study did not involve commercial interests or the use of personal patient data, so no informed consent requirement was required.

The inclusion criterion was a first-time diagnosis of primary lung cancer confirmed by histopathological examination. The exclusion criteria included (1) patients with other malignancies, (2) lung cancer with multiple pathological types, (3) patients who could not be precisely classified according to pathological types, (4) patients lacking results for the five lung cancer markers recommended by the guidelines, and (5) patients without any routine laboratory results. As this was a retrospective epidemiological study, no formal sample size calculation was performed. Instead, all eligible patients were enrolled to maximize statistical power.

### Data Collection and Definition

Data collected included the first routine laboratory tests conducted within 24 h of patient admission and the first set of tumor marker measurements prior to treatment. To minimize biases caused by missing data, variables with more than 20% missing data were excluded from the analysis. Missing clinical features were assumed to occur randomly, and multiple imputation was performed using the random forest method within the multivariate imputation by chained equations package.

Comprehensive clinical data were gathered, encompassing demographic details (sex and age); lung cancer diagnosis and staging information (pathological subtype, TNM, and cTNM classifications); laboratory tests (white blood cell count, hemoglobin, red blood cell distribution width, lymphocyte count, monocyte count, neutrophil count, alanine aminotransferase, aspartate aminotransferase, γ-glutamyl transferase, direct bilirubin, indirect bilirubin, total bilirubin, alkaline phosphatase, total protein, globulin, albumin, albumin-globule ratio, creatinine, blood urea nitrogen, uric acid, total cholesterol, glucose, thrombin time, prothrombin time, thrombin time ratio, international normalized ratio, activated partial thromboplastin time (APTT), APTT ratio, fibrinogen, D-dimer, chloride, calcium, sodium, potassium, magnesium, phosphorus); and lung cancer biomarkers (CEA, CYFRA21-1, SCCA, NSE, ProGRP). Tumor markers were assessed in accordance with the manufacturer's instructions.

The study’s outcome was defined as the pathological classification of lung cancer, encompassing SCLC, SCC, and ADC.

### Statistical Analysis

Continuous variables were presented as either means with standard deviations or medians with interquartile ranges, and categorical variables were reported as absolute values and proportions. All analyses were conducted in R version 4.4.1, with *p* values < 0.05 considered statistically significant.

The cohort was stratified and randomly divided into the training set (70% of the samples) and the test set (30% of the samples) to ensure consistent distribution of lung cancer subtypes across both subsets. Within the training set, the study outcome served as the predicted variable. Key features were selected from all baseline variables using the least absolute shrinkage and selection operator (LASSO) regression and the Boruta algorithm. Features identified by both algorithms were selected as the final model features.^15^

Based on previous research^16,17^ and recognizing that tree-based models often outperform neural networks in processing tabular data,^18^ a logistic regression model was constructed alongside ensemble learning models using decision trees as the base classifier. These ensemble models comprised extreme gradient boosting (XGBoost), categorical boosting (CatBoost), and random forest (RandomForest). Subsequently, a five-fold cross-validation technique was employed to divide the entire dataset into five subsets. Four subsets were used to train the model, and the fifth subset served as an internal validation set for model evaluation. This procedure was repeated five times to optimize the model's hyperparameters. The models' performance was assessed and compared using the area under the receiver operating characteristic curve (AUC), accuracy, and F1 score to identify the most effective model.

To address random forest interpretability, two established metrics were utilized: increase in node purity (IncNodePurity) and percentage of increase in mean square error (%IncMSE). These metrics quantify each feature's contribution to prediction accuracy, providing transparent insights into model decision-making despite the ensemble nature of the algorithm. Specifically, %IncMSE reflects how much the model's prediction error increases when a predictor's values are randomly shuffled, with more important predictors causing a larger increase. IncNodePurity is measured by the sum of squares of the residuals, representing the effect of each variable on the heterogeneity of the observations on each node of the classification tree, thus comparing the importance of the variables. Therefore, higher %IncMSE and IncNodePurity indicate higher feature importance.

Subsequently, the model with the best performance was subjected to independent validation using the test set. The model's generalizability was evaluated using the AUC, accuracy, and F1 score.

Furthermore, an online prediction calculator was developed using the R Shiny framework. This tool facilitates real-time individualized predictions based on clinician-provided parameters, thereby offering intuitive, data-driven support for clinical decision-making.

## Results

### Characteristics of the Study Cohort

**Fig. 1:**
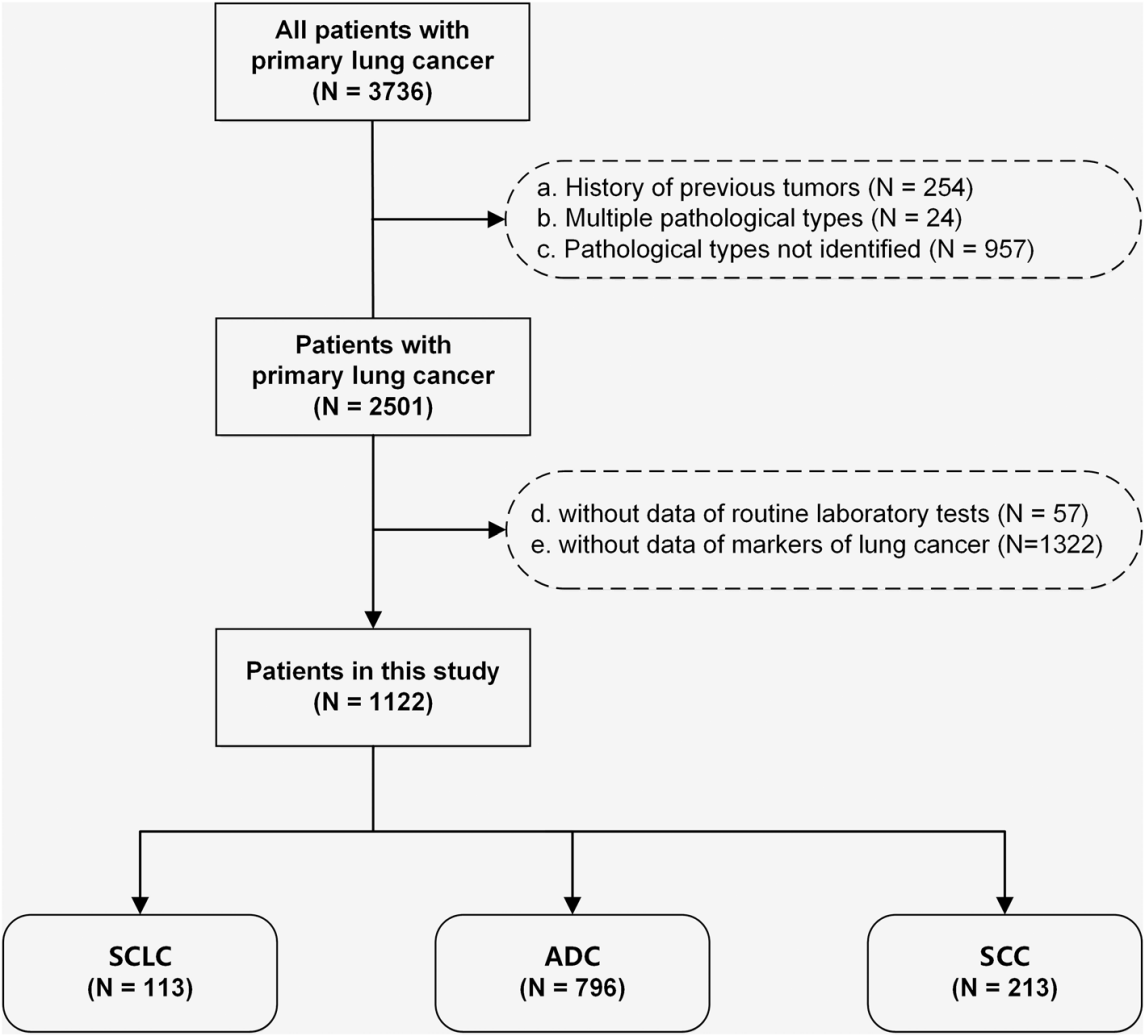
Study flow chart with the inclusion and exclusion criteria of the study. ADC, adenocarcinoma; SCC, squamous cell carcinoma; SCLC, small-cell lung cancer

A total of 3736 patients were diagnosed with primary lung cancer. Based on the established inclusion and exclusion criteria, 1122 patients were selected for the main analysis (Table 1), and 119 patients were selected for the supplemental cohort (Supplemental Table 1). Figure 1 displays the basic information of the study population. In the main analysis, the distribution of patients was as follows: ADC 70.9%, SCC 19.0%, and SCLC 10.1%. The proportion of males was higher in patients with SCLC and SCC, whereas the proportion of females was higher among patients with ADC. Patients with SCLC had higher levels of NSE and ProGRP than those with NSCLC. Additionally, patients with SCC had higher levels of CYFRA21-1 than those with SCLC and ADC.

**Table 1:** Clinical information of the study population

Characteristics	Overall, *N* = 1122	Train, *N* = 787	Test, *N* = 335	*p* Value
Age (years)	59.73 ± 10.39	59.57 ± 10.37	60.11 ± 10.46	0.429
Sex (female)	490 (43.7)	340 (43.2)	150 (44.8)	0.627
Hb (g/L)	129.81 ± 16.93	129.93 ± 16.99	129.55 ± 16.84	0.735
RBC (10^12^/L)	4.33 ± 0.56	4.33 ± 0.57	4.32 ± 0.54	0.772
MCV (fL)	91.74 ± 6.12	91.70 ± 5.81	91.83 ± 6.81	0.757
MPV(fL)	10.73 ± 1.73	10.71 ± 1.76	10.76 ± 1.67	0.700
MCHC (g/L)	327.87 ± 9.77	327.93 ± 9.70	327.73 ± 9.93	0.760
RDW_SD (fL)	43.89 ± 3.71	43.89 ± 3.60	43.90 ± 3.95	0.969
RDW_CV (%)	13.30 (12.70–13.90)	13.30 (12.80–14.00)	13.20 (12.60–13.90)	0.156
WBC (10^9^/L)	5.63 (4.62–7.13)	5.69 (4.69–7.25)	5.39 (4.51–6.78)	0.014
NEU (10^9^/L)	3.62 (2.72–4.89)	3.67 (2.79–5.13)	3.33 (2.64–4.65)	0.004
LYM (10^9^/L)	1.44 ± 0.54	1.45 ± 0.55	1.43 ± 0.52	0.566
MON (10^9^/L)	0.36 (0.28–0.49)	0.37 (0.28–0.50)	0.35 (0.27–0.47)	0.016
BAS (10^9^/L)	0.03 (0.02–0.04)	0.03 (0.02–0.04)	0.03 (0.02–0.04)	0.625
EOS (10^9^/L)	0.11 (0.06–0.20)	0.11 (0.06–0.20)	0.12 (0.07–0.20)	0.220
PLT (10^9^/L)	209.53 ± 79.60	212.24 ± 83.16	203.15 ± 70.23	0.061
PDW	16.26 ± 0.41	16.25 ± 0.42	16.27 ± 0.39	0.559
ALT (U/L)	19.00 (14.00–28.75)	19.00 (14.00–29.00)	19.00 (13.00–26.50)	0.107
AST (U/L)	20.00 (16.00–24.00)	20.00 (16.00–25.00)	19.00 (16.00–23.50)	0.109
ALP (U/L)	82.00 (67.00–100.00)	82.00 (67.00–101.00)	81.00 (67.00–99.00)	0.349
GGT (U/L)	25.00 (17.00–42.00)	25.00 (17.00–43.00)	24.00 (17.00–37.00)	0.340
TBil (umol/L)	10.04 ± 4.48	10.00 ± 4.45	10.12 ± 4.56	0.703
IBil (umol/L)	7.71 ± 3.87	7.70 ± 3.81	7.75 ± 4.01	0.834
DBil (umol/L)	2.31 ± 1.18	2.29 ± 1.22	2.33 ± 1.08	0.615
TP (g/L)	67.15 ± 6.29	67.18 ± 6.33	67.07 ± 6.20	0.779
Alb (g/L)	40.16 ± 4.40	40.17 ± 4.42	40.12 ± 4.36	0.839
PAB (mg/L)	227.97 ± 66.05	229.83 ± 68.09	223.60 ± 60.87	0.131
Glob (g/L)	27.00 ± 4.54	27.01 ± 4.55	26.96 ± 4.53	0.866
A/G ratio	1.53 ± 0.30	1.53 ± 0.29	1.53 ± 0.30	0.806
α-L-Fuc (U/L)	25.18 ± 6.80	25.13 ± 6.85	25.31 ± 6.68	0.672
Cr (umol/L)	72.68 ± 25.37	73.36 ± 28.72	71.07 ± 14.65	0.079
Ccr (ml/min/1.73 m^2^)	90.01 ± 13.94	89.68 ± 14.23	90.77 ± 13.24	0.217
BUN (mmol/L)	5.60 ± 1.62	5.62 ± 1.61	5.54 ± 1.62	0.421
UA (umol/L)	319.47 ± 87.69	321.20 ± 90.56	315.41 ± 80.53	0.289
TC (mmol/L)	4.64 ± 0.97	4.65 ± 0.98	4.61 ± 0.96	0.483
Glu (mmol/L)	5.05 (4.65–5.70)	5.06 (4.66–5.68)	5.00 (4.64–5.76)	0.314
Cl (mmol/L)	105.98 ± 3.41	105.88 ± 3.45	106.20 ± 3.31	0.155
Ca (mmol/L)	2.28 ± 0.13	2.29 ± 0.13	2.27 ± 0.13	0.033
Na (mmol/L)	140.99 ± 3.04	140.93 ± 3.02	141.11 ± 3.08	0.374
K (mmol/L)	4.12 ± 0.36	4.12 ± 0.36	4.10 ± 0.36	0.457
Mg (mmol/L)	0.87 ± 0.08	0.87 ± 0.08	0.88 ± 0.08	0.137
P (mmol/L)	1.11 ± 0.18	1.12 ± 0.17	1.09 ± 0.18	0.020
TT (seconds)	17.34 ± 1.79	17.35 ± 2.03	17.32 ± 1.05	0.774
PT (seconds)	12.15 ± 1.33	12.14 ± 1.33	12.16 ± 1.32	0.804
TTR	1.03 ± 0.10	1.03 ± 0.12	1.03 ± 0.06	0.689
INR	0.96 ± 0.10	0.96 ± 0.10	0.96 ± 0.10	0.721
APTT (seconds)	31.45 ± 6.47	31.21 ± 6.49	32.01 ± 6.40	0.057
APTTR	1.09 ± 0.22	1.08 ± 0.22	1.11 ± 0.22	0.017
Fibrinogen (g/L)	3.87 ± 1.59	3.90 ± 1.65	3.82 ± 1.44	0.398
FDP (μg/ml)	2.40 (1.80–3.50)	2.40 (1.80–3.50)	2.30 (1.80–3.35)	0.867
DD (μg/ml)	0.70 (0.54–1.10)	0.70 (0.54–1.14)	0.69 (0.55–0.96)	0.651
CEA (ng/ml)	2.32 (1.34–4.40)	2.36 (1.36–4.44)	2.17 (1.31–4.21)	0.276
CYFRA21_1 (ng/ml)	2.78 (1.93–4.99)	2.79 (1.93–5.04)	2.75 (1.97–4.94)	0.954
SCCA (ng/ml)	0.75 (0.56–1.06)	0.73 (0.56–1.07)	0.77 (0.56–1.03)	0.708
NSE (ng/ml)	13.29 (11.13–17.53)	13.36 (11.33–17.67)	12.99 (10.88–17.16)	0.229
ProGRP (pg/ml)	43.00 (34.00–56.81)	43.28 (34.26–56.59)	42.58 (33.49–57.19)	0.322
CA19_9 (U/ml)	12.92 (7.56–21.75)	12.57 (7.37–21.51)	13.28 (7.93–24.09)	0.323
Lung cancer				0.440
SCLC	113 (10.1)	85 (10.8)	28 (8.4)	
ADC	796 (70.9)	552 (70.1)	244 (72.8)	
SCC	213 (19.0)	150 (19.1)	63 (18.8)	

Data are presented as n (%), mean ± standard deviation, or median (interquartile range) unless otherwise indicated.

*α-L-Fuc* alpha L fucosidase creatinine, *A/G ratio* albumin-globulin ratio, *ADC* adenocarcinoma, *Alb* albumin, *ALP* alkaline phosphatase, *ALT* alanine aminotransferase, *APTT* activated partial thromboplastin time, *APTTR* APTT ratio, *AST* aspartate aminotransferase, *BAS* basophils, *BUN* blood urea nitrogen, *Ca* calcium, *CA19-9* carbohydrate antigen 19-9, Ccr creatinine clearance rate, *CEA* carcinoembryonic antigen, *Cl* chlorine, *Cr* creatinine, *CYFRA21-1* cytokeratin fragment 21-1, *DBil* direct bilirubin, *DD* D-dimer, *EOS* eosinophils, *FDP* fibrin degradation products, *GGT* gamma glutamyl transferase, *Glob* globulin, *Glu* glucose, *Hb* hemoglobin, *IBil* indirect bilirubin, *INR* international normalized ratio, *K* potassium, *LYM* lymphocytes, *MCHC* mean corpuscular hemoglobin concentration, *MCV* mean corpuscular volume, *Mg* magnesium, *MON* monocytes, *MPV* mean platelet volume, *Na* sodium, *NEU* neutrophils, *NSE* neuron-specific enolase, *P* phosphorus, *PAB* prealbumin, *PDW* platelet distribution width, *PLT* platelets, *ProGRP* pro-gastrin-releasing peptide, *PT* prothrombin time, *RBC* red blood cell, *RDW* RBC distribution width, *SCCA* squamous cell carcinoma antigen, *SCLC* small-cell lung cancer, *SCC* squamous cell carcinoma, *TBil* total bilirubin, *TC* total cholesterol, *TP* total protein, *TT* thrombin time, *TTR* thrombin time ratio, *UA* uric acid, *WBC* white cell count

### Feature Selection

**Fig. 2:**
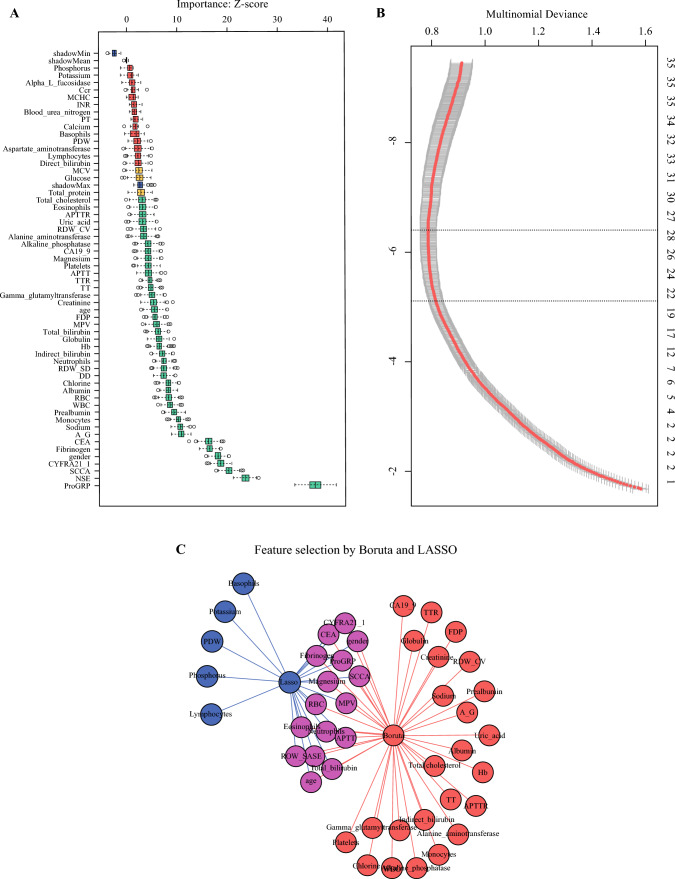
Feature selection based on the Boruta and least absolute shrinkage and selection operator (LASSO) algorithm. Clinical feature selection using Boruta methods (**A**). LASSO algorithm outputted filtered features with non-zero coefficients (**B**). Sixteen features came from the intersection of the two algorithms (**C**). APTT, activated partial thromboplastin time; APTTR, APTT ratio; CA19-9, carbohydrate antigen 19-9; CEA, carcinoembryonic antigen; DD, D-dimer; FDP, fibrin degradation products; Hb, hemoglobin; INR, international normalized ratio; MCHC, mean corpuscular hemoglobin concentration; MCV, mean corpuscular volume; MPV, mean platelet volume; NSE, neuron-specific enolase; PDW, platelet distribution width; ProGRP, pro-gastrin-releasing peptide; PT, prothrombin time; RDW, red blood cell distribution width; SCCA, squamous cell carcinoma antigen; TT, thrombin time; TTR, thrombin time ratio; WBC, white cell count

The LASSO and Boruta algorithms identified 16 features relevant to various lung cancer subtypes. These features included sex, age, total bilirubin, red blood cells, mean platelet volume, red blood cell distribution width (RDW)_SD, neutrophils, eosinophils, fibrinogen, magnesium, APTT, CEA, CYFRA21-1, SCCA, NSE, and proGRP (Fig. 2).

### Model construction and interpretability

**Fig. 3:**
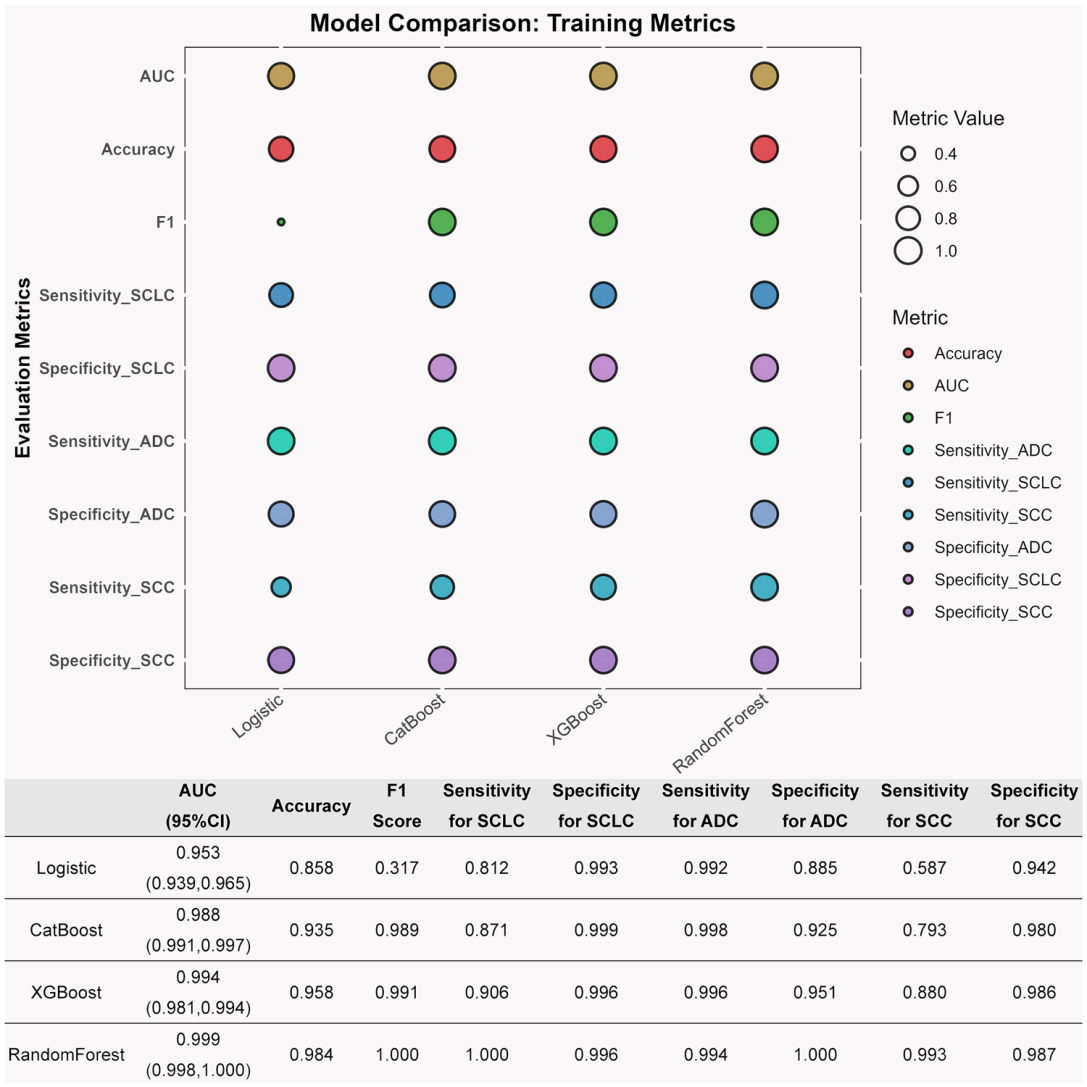
Diagnostic performance of four algorithmic models in training set. ADC, adenocarcinoma; AUC, area under the curve; SCC, squamous cell carcinoma; SCLC, small-cell lung cancer

Logistic regression and ensemble algorithm models were developed using the above features within the training set. Subsequently, the diagnostic capabilities of the logistic regression model and the RandomForest, XGBoost, and CatBoost models, each optimized with hyperparameters, were compared directly within the training set. The analysis indicated that the RandomForest model exhibited superior accuracy, so it was selected as the optimal model, with an AUC of 0.999, an accuracy of 0.984, and an F1 score of 1.000 (Fig. 3).

**Fig. 4:**
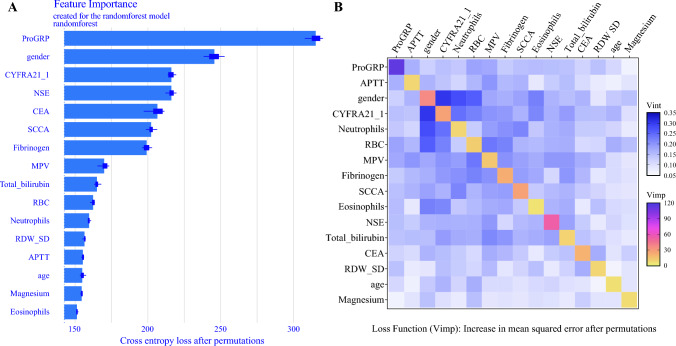
Visualization of the importance ranking of predictive features of RandomForest model. APTT, activated partial thromboplastin time; CEA, carcinoembryonic antigen; MPV, mean platelet volume; NSE, neuron-specific enolase; ProGRP, pro-gastrin-releasing peptide; RBC, red blood cells; RDW, RBC distribution width; SCCA, squamous cell carcinoma antigen

Furthermore, the %IncMSE and IncNodePurity metrics were utilized to evaluate feature importance within the RandomForest model. The analysis revealed that sex, CEA, CYFRA21-1, SCCA, NSE, and proGRP showed the greatest contribution to model development and outcome prediction (Fig. 4).

### Discriminative power and online deployment of the model

**Fig. 5:**
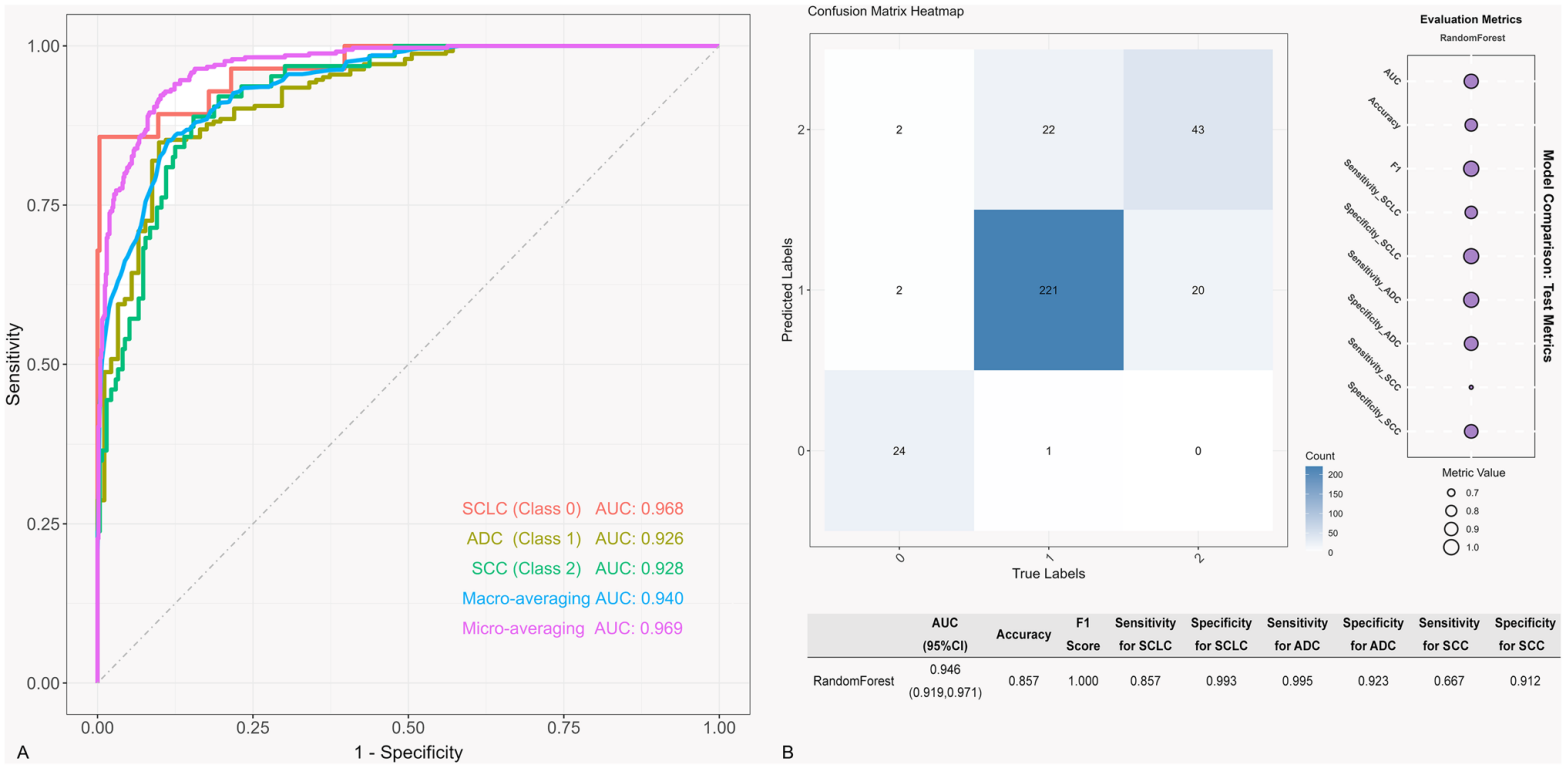
Diagnostic performance of RandomForest model in test set. ADC, adenocarcinoma; AUC, area under the curve; CI, confidence interval; SCC, squamous cell carcinoma; SCLC, small-cell lung cancer

The model's predictive performance was evaluated using the test set. The receiver operating characteristic curves for the RandomForest model in predicting lung cancer subtypes demonstrated micro- and macro-averaged AUCs of 0.969 and 0.940, respectively (Fig. 5A). Following the model's predictions, probabilities were generated for three potential subtypes, with the subtype exhibiting the highest probability designated as the predicted outcome. Consequently, the sensitivity and specificity for the predicted subtypes—SCLC, SCC, and ADC—were 0.857 and 0.993, 0.995 and 0.923, and 0.667 and 0.912, respectively (Fig. 5B).

In addition, to further evaluate the contribution of the top six features, additional models were constructed using these six features as predictors, driven by the same hyperparameters. This concise version of the model achieved strong performance in the test set, with micro- and macro-averaged AUCs of 0.943 and 0.899, respectively (Supplementary Figure 1). The model also achieved respectable performance in the supplemental cohort, with micro- and macro-averaged AUCs of 0.878 and 0.856, respectively (Supplementary Figure 2). These findings support the interpretability analysis results.

**Fig. 6:**
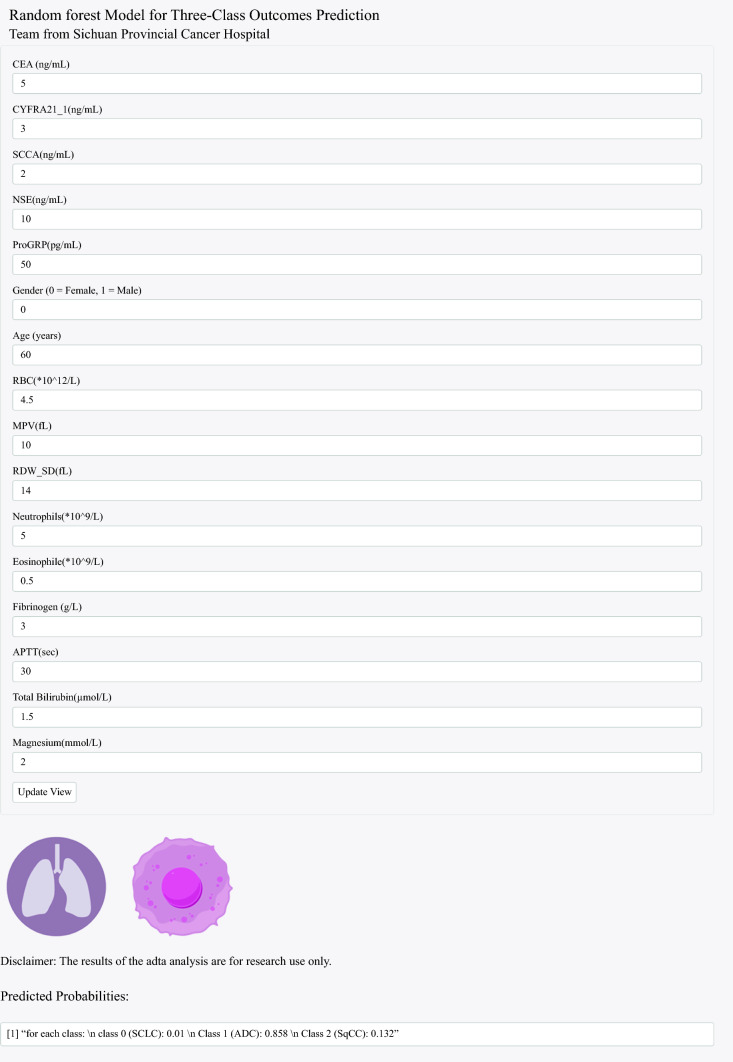
Diagram of online calculator for classification in lung cancer. Input the information and click the “Update View” button to get the patient’s assessment results. APTT, activated partial thromboplastin time; CEA, carcinoembryonic antigen; MPV, mean platelet volume; NSE, neuron-specific enolase; proGRP, pro-gastrin-releasing peptide; RBC, red blood cells; RDW, RBC distribution width; SCCA, squamous cell carcinoma antigen

A web-based calculator was developed based on the RandomForest model to facilitate model application (https://nkuwangkai.shinyapps.io/lung-cancer-v1/). This online calculator automatically calculates the probability predictions upon entering values of the 16 predefined parameters (Fig. 6).

## Discussion

In this study, a machine learning-based model was developed using retrospective cohort data to investigate the prediction of various lung cancer subtypes. The RandomForest algorithm demonstrated excellent predictive performance in distinguishing lung cancer subtypes, achieving a micro-averaged AUC of 0.969. Key features identified using the LASSO and Boruta algorithms included sex, CEA, CYFRA21-1, SCCA, NSE, and ProGRP. These biomarkers and clinical characteristics are associated with lung cancer subtypes and provide key insights for noninvasive characterization of the disease.

The treatment of lung cancer depends heavily on precise staging and classification. For instance, surgical radical resection is the preferred therapeutic approach for patients with stage II and II NSCLC, whereas systemic treatment is indicated for stage IV disease after determining the NSCLC pathological type. Nonetheless, it can be challenging to undertake a comprehensive histopathological evaluation in certain patients, complicating the treatment process. The American Cancer Society National Roundtable on Lung Cancer has highlighted inconsistencies in the guidelines for lung cancer staging. It has recommended that comparative effectiveness studies be prioritized to evaluate cancer staging practices and to promote unifying guideline recommendations across various professional societies.^19^ These initiatives aim to enhance the consistency and accuracy of lung cancer staging, which is crucial for delivering stage-appropriate treatment. However, such improvements do not fully address the challenges in completing histopathological assessments. The development of complex algorithms, such as those based on machine learning, may offer alternative methods for more accurate cancer assessment. Notably, recent progress has shown the translational potential of integrating clinical features with machine learning techniques in lung cancer research. One study introduced an innovative machine learning-based model for lung cancer screening and staging, utilizing routine clinical and laboratory information, effectively enhancing diagnostic accuracy.^20^ Similarly, another study analyzed standard blood samples and smoking history data from high-risk populations to develop a machine learning model employing dynamic ensemble selection for the early detection of lung cancer.^21^ Additionally, a study emphasized the application of a machine learning model for the early prediction of lung cancer invasion, integrating enhanced computed tomography (CT) features and serum biomarkers of lung nodules, achieving accuracies of 0.857 and 0.955 in the validation and test groups, respectively.^22^ Collectively, these investigations contrast with omics approaches, which have rapidly advanced in recent years within the domain of lung cancer evaluation. Considering the lack of specific predictive tools for lung cancer classification, the early stage of current evaluation processes, and the substantial labor and economic costs associated with omics technologies, there is a growing demand for more practical tools. The predictive model proposed in this study, tailored to the clinical context, offers a robust tool for devising non-invasive treatment strategies and facilitating risk communication and shared decision-making between clinicians and patients.

With a deeper understanding of lung cancer biology, the integration of tumor markers into clinical practice remains essential for optimizing patient care and enhancing therapeutic strategies. In this study, advanced feature selection methodologies were employed to identify key features. The interpretation of these model features rendered the traditionally opaque "black box" model more transparent. Importantly, readily available clinical features and tumor markers possess significant biological relevance, as opposed to radiomics. Notably, these features were calculated using the RandomForest algorithm and have demonstrated strong predictive capabilities. The performance of our model is also comparable to existing lung cancer classification methods. For example, Ye et al.^23^ achieved lung cancer prediction with an AUC of about 0.77 based on clinical features and logistic regression methods. Ardila et al.^24^ achieved lung cancer prediction with an AUC of about 0.95 based on CT radiomics and deep learning.^25^ Moreover, translating machine learning models into clinically practical solutions presents a novel challenge. Despite the robust computational capabilities of machine learning algorithms, only a small number of studies have developed websites or applications to enhance their usability.^26^ In response, our team developed a web-based calculator that presents the predicted outcomes of subtype classification. Continuous updates and further validation would facilitate its integration into personalized clinical practice. Meanwhile, our research focuses on routine clinical and laboratory markers as these indices are readily available in resource-limited settings, do not require specialized equipment, and are cost effective for widespread implementation. However, liquid biopsy techniques, including circulating tumor DNA and circulating tumor cells, represent promising biomarkers for lung cancer classification.^27,28^ Future iterations of our model could incorporate liquid biopsy markers, potentially improving predictive accuracy while maintaining a foundation of readily available clinical parameters.

The predominance of adenocarcinoma in our cohort (70.9% ADC, 19.0% SCC, 10.1% SCLC) reflects the epidemiological reality of lung cancer distribution in contemporary clinical practice. Recent epidemiological studies have shown that adenocarcinoma now accounts for 60–75% of NSCLC cases globally, representing a shift from historical patterns where SCC was more prevalent.^29^ This distribution is particularly pronounced in Asian populations and among never-smokers,^30^ which aligns with our hospital's patient demographics. Although the class imbalance could theoretically bias our model toward better performance in adenocarcinoma prediction, several strategies were implemented to mitigate this concern: stratified random splitting was employed to ensure proportional representation across training and test sets; the RandomForest algorithm inherently handles class imbalance through bootstrap sampling and ensemble voting; performance was evaluated using both micro- and macro-averaged metrics, where macro-averaging gives equal weight to each class regardless of support, providing a more balanced assessment of model performance across all subtypes. Notably, despite the class imbalance, our model achieved robust performance for minority classes, with a sensitivity of 0.857 for SCLC and 0.995 for SCC, demonstrating that the model learned meaningful discriminative features rather than simply defaulting to the majority class. The high importance of subtype-specific markers (ProGRP/NSE for SCLC, SCCA/CYFRA21-1 for SCC) in our feature importance analysis further supports this interpretation. Nevertheless, we acknowledge this imbalance as a limitation. Future multicenter studies should prioritize enrichment of minority subtypes through targeted recruitment or synthetic data augmentation techniques to ensure robust performance across all lung cancer subtypes.

In addition, biopsy procedures may be constrained by inter- and intra-tumoral heterogeneity, potentially resulting in incomplete descriptions of tumor morphology and phenotype,^31^ particularly in institutions where histopathological evaluation is still in its early stages. Although molecular tests can identify specific driver mutations for precision medicine, their integration with routine diagnostics is often limited by the expertise required and the high costs. Given the high predictive performance of the model proposed in this study, there is potential for future enhancements to assist in the screening of lung cancer subtypes. Moreover, during the treatment of lung cancer, patients with ADC harboring EGFR-sensitive mutation genes that exhibit resistance to EGFR-TKI therapy may undergo transformation to SCLC, necessitating a change in treatment strategy. However, frequent biopsies can impose a significant burden on patients. In this context, leveraging the high predictive performance model proposed in this study presents a viable alternative. The model enables the updating of lung cancer subtype classification predictions based on the most recent clinical features and tumor marker information. Overall, this approach may improve diagnostic capabilities in underdeveloped regions.

Nevertheless, the limitations of this study should be acknowledged. First, the retrospective design introduces potential selection bias, as only patients with complete tumor marker panels were included, potentially excluding patients with advanced disease or poor performance status. To partially mitigate this, multiple imputation of missing routine laboratory test values was performed. The unicentric nature limits generalizability, as patient demographics, diagnostic practices, and tumor marker testing platforms can vary by institution. A supplemental cohort was utilized for initial external validation, achieving a micro- and macro-averaged AUC of 0.878 and 0.856, respectively. Still, comprehensive multicenter validation remains critical. In addition, the model was deployed as a web-based application to facilitate external validation and confirm its robustness. Second, although the total cohort comprised 1122 patients, the proportions of SCLC (10.1%) and SCC (19.0%) were relatively small compared with ADC (70.9%), potentially impacting the model's performance for these minority subtypes. Future studies should prioritize enrichment of a few subtypes through targeted recruitment to ensure robust performance of all lung cancer subtypes. Third, the model's predictive capability for dynamic changes in tumor subtypes, such as the transformation of EGFR-mutant NSCLC into SCLC, remains untested. These represent a prospective direction for our future research endeavors.

## Conclusions

This study provides evidence supporting the application of machine learning in predicting pathological subtypes in patients with lung cancer. By identifying key clinical predictors and integrating them into a machine learning framework, a RandomForest prediction model was developed to help clinicians identify different subtypes of lung cancer under non-puncture conditions. This may be beneficial for patients who are unable to complete a histopathologic evaluation. Further validation and refinement of the model in different clinical settings is essential to establish its practical application and optimize patient care outcomes.

## Supplementary Information

Below is the link to the electronic supplementary material.

Supplementary file1 (DOCX 27 KB)

Supplemental figure 1. Diagnostic performance of the concise Random Forest model in test set driven by the same hyperparameters (TIF 49004 KB)

Supplemental figure 2 Diagnostic performance of the concise Random Forest model in the supplementary cohort driven by the same hyperparameters (TIF 47296 KB)

## Data Availability

The datasets presented in this study can be obtained from the corresponding author upon reasonable request.
