# Supplementary material for: Machine Learning for Classification in Lung Cancer Using Routine Clinical and Laboratory Data
Source: Ann Surg Oncol. 2025 Dec 3;33(4):3100–12. doi: 10.1245/s10434-025-18747-y (PMC12982216; doi:10.1245/s10434-025-18747-y)
Supplement: Supplementary file 1 — Supplementary file1 (DOCX 27 KB) [file 10434_2025_18747_MOESM1_ESM.docx]

**Supplemental table 1.** Clinical information of the supplementary cohort.

| **Characteristics** | **Overall, N = 119** | **SCLC, N = 9** | **ADC, N = 76** | **SqCC, N = 34** | ***p* value** |
| --- | --- | --- | --- | --- | --- |
| Gender (female) | 54 (45.4%) | 4 (44.4%) | 46 (60.5%) | 4 (11.8%) | < 0.001 |
| CEA(ng/ml) | 2.58 (1.71, 10.94) | 1.99 (1.94, 4.06) | 3.27 (1.66, 18.04) | 2.56 (1.80, 4.54) | 0.510 |
| CYFRA21_1(ng/ml) | 4.40 (2.30, 9.90) | 3.90 (1.80, 4.41) | 3.05 (2.05, 6.60) | 9.75 (4.40, 18.50) | < 0.001 |
| SCCA(ng/ml) | 0.99 (0.60, 2.00) | 0.59 (0.45, 0.95) | 0.85 (0.56, 1.40) | 3.28 (1.58, 5.25) | < 0.001 |
| NSE(ng/ml) | 12.51 (8.91, 15.93) | 19.26 (18.62, 161.60) | 11.83 (8.18, 15.50) | 12.87 (9.72, 15.84) | 0.019 |
| ProGRP(pg/ml) | 44.65 (30.34, 66.72) | 953.50 (2.99, 1121.00) | 43.02 (29.65, 66.52) | 47.64 (38.08, 64.42) | 0.305 |

CEA: Carcinoembryonic Antigen. CYFRA21-1: Cytokeratin Fragment 21-1. SCCA: Squamous Cell Carcinoma Antigen. NSE: Neuron Specific Enolase . ProGRP: Pro-Gastrin-Releasing Peptide . SCLC: Small Cell Lung Cancer. ADC: Adenocarcinoma. SqCC: Squamous Cell Carcinoma.
