# Supplementary figures and images for: Machine Learning for Classification in Lung Cancer Using Routine Clinical and Laboratory Data
Source: Ann Surg Oncol. 2025 Dec 3;33(4):3100–12. doi: 10.1245/s10434-025-18747-y (PMC12982216; doi:10.1245/s10434-025-18747-y)

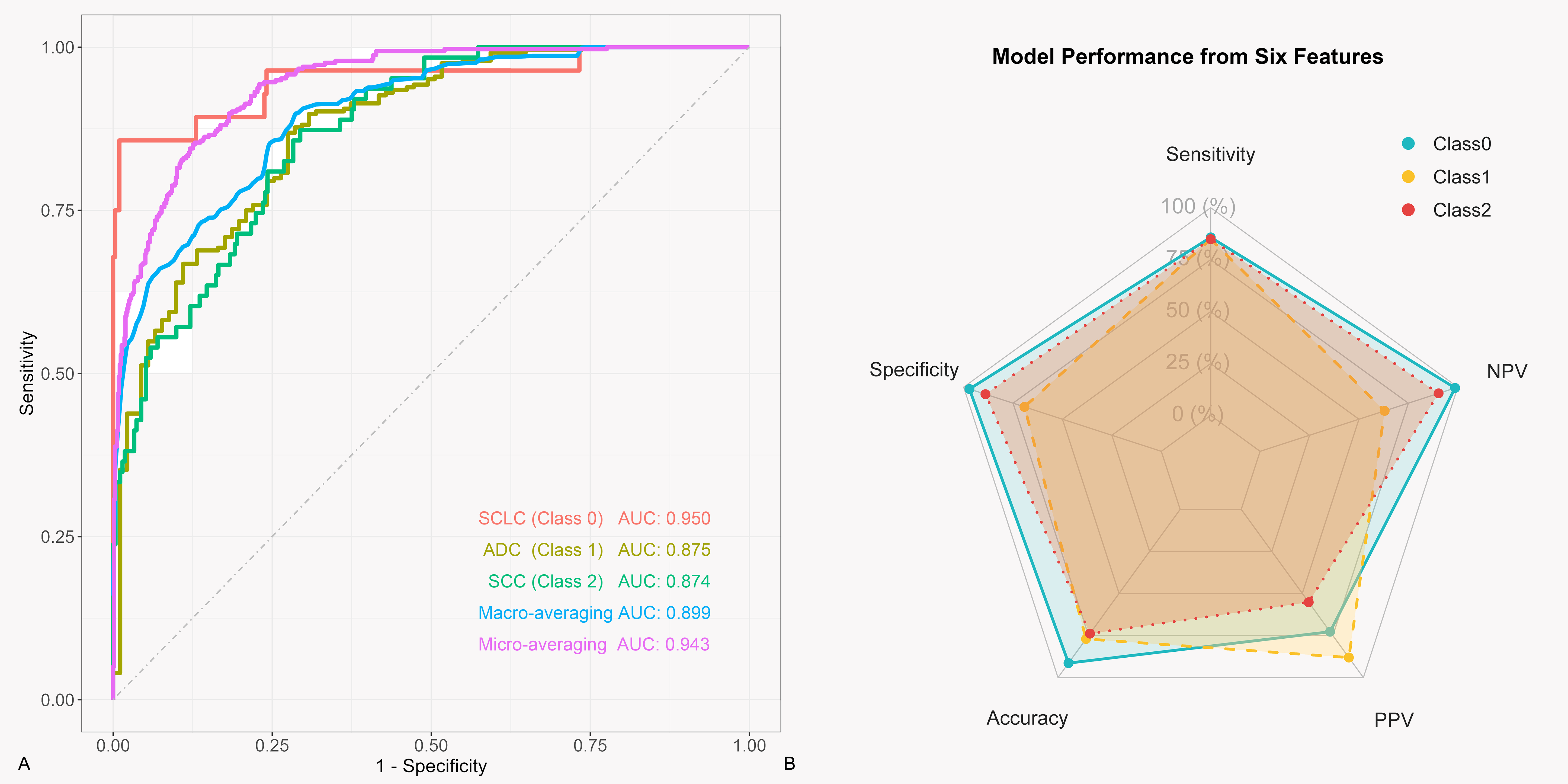

Supplement: Supplementary file 2 — Supplemental figure 1. Diagnostic performance of the concise Random Forest model in test set driven by the same hyperparameters (TIF 49004 KB) [file 10434_2025_18747_MOESM2_ESM.tif]

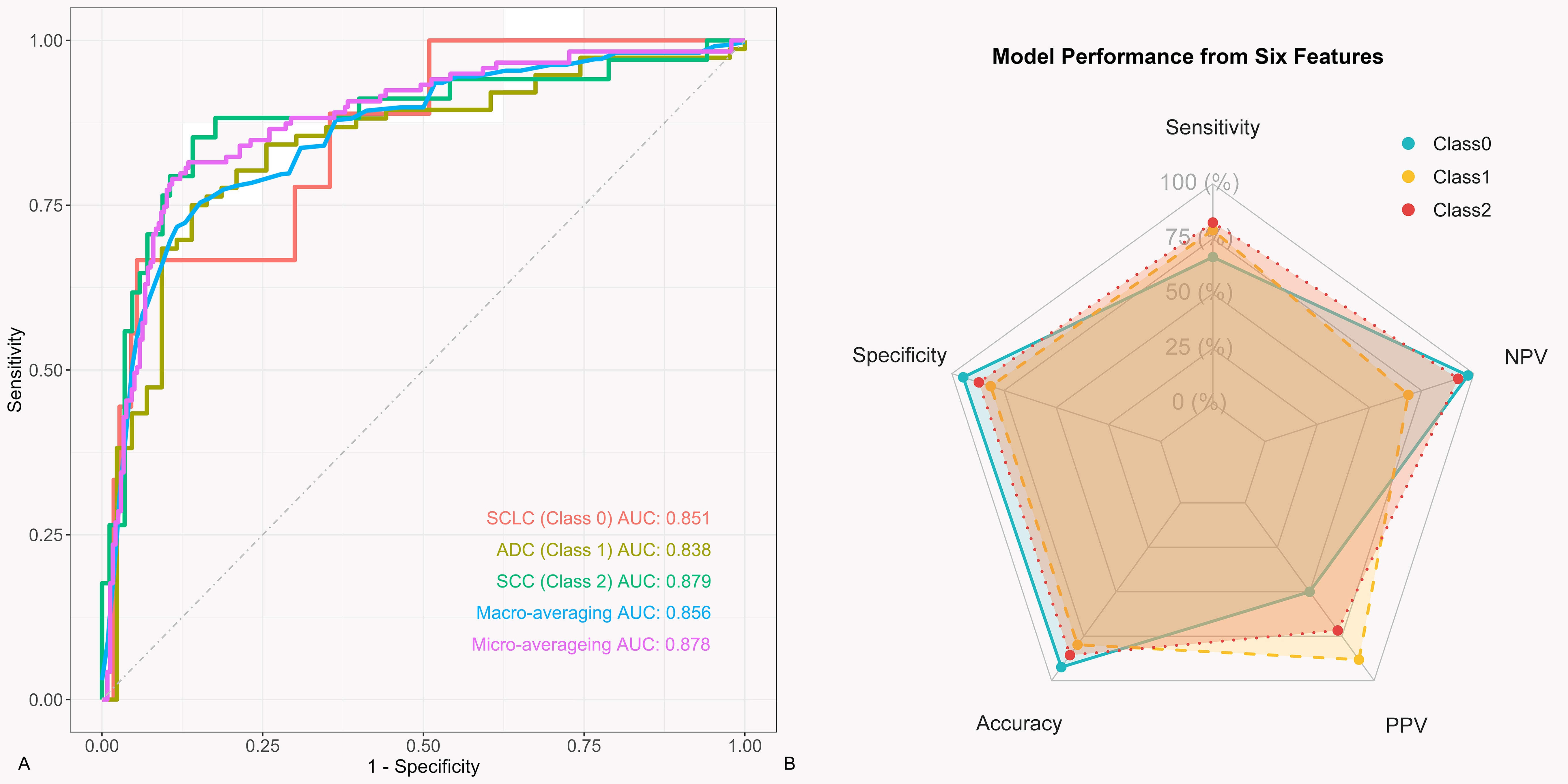

Supplement: Supplementary file 3 — Supplemental figure 2 Diagnostic performance of the concise Random Forest model in the supplementary cohort driven by the same hyperparameters (TIF 47296 KB) [file 10434_2025_18747_MOESM3_ESM.tif]
